# The Role of Environmental Factors in Technology-Assisted Physical Activity Intervention Studies Among Older Adults: Scoping Review

**DOI:** 10.2196/59570

**Published:** 2025-03-13

**Authors:** Carl-Philipp Jansen, Désirée Nijland, Jean-Michel Oppert, Veysel Alcan, Kirsi E Keskinen, Emmi Matikainen-Tervola, Zada Pajalic, Merja Rantakokko, Signe Tomsone, Essi-Mari Tuomola, Erja Portegijs, Erik J Timmermans

**Affiliations:** 1 Geriatric Center Medical Faculty Heidelberg Heidelberg University Heidelberg Germany; 2 Clinic for Geriatric Rehabilitation Robert Bosch Hospital Stuttgart Germany; 3 Department of Human Movement Sciences University Medical Center Groningen University of Groningen Groningen The Netherlands; 4 Department of Nutrition Pitié-Salpêtrière Hospital (AP-HP) Sorbonne University Paris France; 5 Department of Electrical and Electronics Engineering Engineering Faculty Tarsus University Tarsus Turkey; 6 Gerontology Research Center and Faculty of Sport and Health Sciences University of Jyväskylä Jyväskylä Finland; 7 Institute of Rehabilitation JAMK University of Applied Sciences Jyväskylä Finland; 8 Campus Drammen Faculty of Health and Social Sciences University of South-Eastern Norway Oslo Norway; 9 The Wellbeing Services County of Central Finland Jyväskylä Finland; 10 Department of Rehabilitation Faculty of Health and Sport Sciences Rīga Stradiņš University Riga Latvia; 11 Julius Center for Health Sciences and Primary Care University Medical Center Utrecht Utrecht University Utrecht The Netherlands

**Keywords:** environmental factors, intervention, older adults, physical activity, technology, PRISMA

## Abstract

**Background:**

The rapidly emerging integration of both technological applications and environmental factors in physical activity (PA) interventions among older adults highlights the need for an overarching investigation.

**Objective:**

This scoping review compiled the current literature and aimed to provide an overview of the role of physical, social, socioeconomic, and systemic environmental factors in technology-assisted PA interventions for older adults.

**Methods:**

We systematically searched 6 common databases up to September 16, 2024, for original longitudinal studies (with at least one preintervention measurement and one postintervention measurement) that reported on the role of environmental factors in technology-assisted PA interventions for independently living, community-dwelling older adults. In a stepwise process, data on study characteristics (step 1), environmental factors and their role in the included studies (step 2), and intervention outcomes and effects by type of environmental factor (step 3) were summarized.

**Results:**

A total of 8020 articles were screened, and 25 (0.31%) were included. Most studies were conducted in Europe (11/25, 44%), followed by North America (5/25, 20%), Asia (5/25, 20%), and Oceania (4/25, 16%). Social environmental factors were most often considered (19/25, 76%), followed by factors from the physical (8/25, 32%), socioeconomic (1/25, 4%), and systemic environment (1/25, 4%). Environmental factors were used as the outcome (8/25, 32%), setting variable (7/25, 28%), moderator or facilitator (8/25, 32%), and intervention component (3/25, 12%). In most studies (19/25, 76%), the intervention had a beneficial effect on the outcome of interest, and the included environmental factor played a supportive role in achieving this effect. In some studies, no effect (3/25, 12%), mixed effects (2/25, 8%), or adverse effects (1/25, 4%) of the interventions were reported.

**Conclusions:**

This is the first comprehensive description of how environmental factors interact with technology-assisted interventions to increase or optimize PA in older adults. It was found that the investigation of environmental factors in this field is at an early stage. Environmental factors were found to play a supportive role in achieving beneficial effects of technology-assisted PA interventions, but the findings were based on a heterogeneous empirical platform. Still, certain aspects such as the application of virtual reality environments and social (or peer) comparison have shown significant potential that remains to be leveraged. A better understanding of intervention results and support in tailoring intervention programs can be provided through the inclusion of environmental aspects in technology-assisted PA interventions for older adults.

## Introduction

### Background

Physical activity (PA) in older adults is crucial for the prevention of major chronic noncommunicable diseases and for the improvement or maintenance of mobility, independence, and quality of life [1-3]. Although the health benefits of PA are well established, the prevalence of insufficient PA among older adults is high and points to a considerable scope and need for improvement [4-7]. Therefore, interventions are needed to encourage older adults to initiate and maintain regular PA [8].

In the past 2 decades, PA interventions have increasingly been incorporating technological applications because these may help increase motivation and adherence among participants, (remotely) measure and monitor (changes in) intervention outcomes, and provide feedback about this to participants [9-11]. Previous reviews and meta-analyses have shown that technological applications such as websites, mobile or wearable devices, smartphone apps, and virtual reality have been reported to support PA in the older adult population [9,11-16].

In parallel, the importance of applying socioecological approaches to advancing the understanding of PA determinants, including those from different environmental domains, has increasingly been acknowledged [8,17]. Previous research has emphasized that a variety of physical, social, socioeconomic, and systemic environmental factors play a crucial role in facilitating or hindering PA in older adults [18-20]. Therefore, environmental factors have also been considered increasingly in PA interventions among older adults [8,17]. For instance, one study indicated that a PA intervention was more effective in maintaining or increasing older adults’ PA when implemented in more walkable neighborhoods that are characterized by higher levels of residential density, land use mix, and intersection density [21]. In addition to characteristics of the physical environment (eg, walkability) [8,21], previous studies have also indicated that aspects of the social (eg, receiving social support) [22], socioeconomic (eg, area-level income) [23], and systemic (eg, ethnicity and climate) [24,25] environment are important to consider when implementing PA interventions among (older) adults. However, environmental factors have rarely been investigated regarding their impact on adherence and effectiveness of technology-assisted PA interventions [10,26]. The rapidly emerging integration of both technological applications and environmental factors in PA interventions in the recent past justifies and at the same time highlights the need for an overarching investigation.

### Objectives

Against this background, this scoping review compiled the current literature and provided an overview of the role of physical, social, socioeconomic, and systemic environmental factors in technology-assisted PA interventions for older adults. In this way, we provided new knowledge on the specific environmental factors that have been considered in such previous interventions and investigated whether these factors are associated with adherence to and outcomes of interventions. Furthermore, we aimed to increase insights into how environmental factors may modify outcomes, how they are affected by technology-assisted PA interventions, or how they might be part of underlying mechanisms of this type of interventions. As a consequence of the work at hand, health care professionals, policy makers, and researchers may be enabled to better design effective technology-assisted PA interventions for the target group of older adults.

## Methods

This scoping review followed the PRISMA-ScR (Preferred Reporting Items for Systematic Reviews and Meta-Analyses Extension for Scoping Reviews; Multimedia Appendix 1) guidelines [27], and the protocol was registered on the Open Science Framework platform in March 2023 [28].

### Literature Search

A search query was carried out on March 16, 2023, and updated on September 16, 2024. It was run in 6 bibliographic databases: CINAHL, Embase, MEDLINE, PsycINFO, Scopus, and Web of Science. The search algorithm was built using search terms based on definitions and synonyms of intervention types; technologies; the target group (ie, older adults); and the physical, social, socioeconomic, and systemic environment and its attributes. The search was not limited to a specific time frame. A detailed search strategy for each bibliographic database can be found in Multimedia Appendix 2. The reference lists of the included articles were screened to identify additional eligible papers.

### Inclusion and Exclusion Criteria

In this scoping review, only published articles in the form of original, longitudinal intervention studies with at least one preintervention measurement and one postintervention measurement and with any health-, function-, or behavior-related outcome were included. Of those articles, only papers were selected that included (1) independently living, community-dwelling older adults aged ≥60 years (ie, study sample average age of ≥60 years with a minimum individual age of 50 years) regardless of their health status; (2) an intervention period involving PA (components); (3) a PA intervention with or without a control setting that was based on or aided or strengthened by technology applications or technological components; (4) an assessment of physical, social, socioeconomic, or systemic environmental factors or a specific comparison of groups with different environmental conditions; and (5) a report of associations of environmental factors with adherence to or outcomes of the PA intervention or whether environmental factors were included as an outcome. The language restriction was set to English, Dutch, German, French, Finnish, Latvian, Norwegian, Swedish, and Turkish because these languages were spoken by the review team. Articles that were literature reviews, study protocols, conference proceedings, or abstracts only were excluded.

### Study Selection

After removing duplicate records in EndNote (version 20.0; Clarivate Analytics), the title and abstract of each record were independently screened by 2 reviewers out of the pool of reviewers (ie, all authors of this scoping review). To increase consistency between reviewers, the procedure was discussed within the group of reviewers, who first screened a set of 30 records as a pilot test and discussed results before initiating the full-text screening. Subsequently, the full text of each potentially relevant paper was independently assessed for eligibility by 2 reviewers. Any disagreement between reviewers was resolved through discussion or, if no consensus could be reached, through discussion with a third reviewer. The screening of titles, abstracts, and full texts was conducted using the Rayyan software (Qatar Computing Research Institute), a noncommercial web-based application [29].

### Data Extraction

The data extraction from each eligible paper was performed by 2 reviewers per manuscript using a predefined standardized extraction form in Microsoft Excel (Microsoft Corp). Both reviewers then compared their results and harmonized their findings. Any data extraction issues identified by the reviewers were resolved through group discussion. For each eligible paper, information was extracted on (1) study characteristics (ie, full reference, country in which the study was conducted, and main study objectives); (2) methodological aspects of the study (ie, sample size, study sample characteristics, and study design); (3) intervention components (eg, PA component, duration and frequency of PA or exercise sessions, and supervision of sessions); (4) technology components (ie, technology devices used); (5) environmental factors (ie, type of environmental factor included, such as physical, social, socioeconomic, or systemic), an assessment tool for environmental factors, specific environmental characteristics assessed, and the role of an environmental factor (eg, outcome, a feature of the study design, or a factor with a modifying effect on the intervention outcome); (6) study outcomes (ie, primary and secondary outcomes, outcome measurements, and main study findings); and (7) main study findings in relation to the environmental factors assessed. The reviewers did not contact the authors of the eligible articles to collect unreported data or additional details.

### Data Analysis

In this scoping review, data from the included studies were analyzed through a stepwise process. First, study characteristics were described using descriptive statistics. Second, the environmental factors and their role in technology-assisted PA interventions were described per environmental domain. Third, intervention outcomes were described by each type of environmental factor, and the actual effects of or on environmental factors were summarized.

## Results

### Literature Search

**Figure 1:**
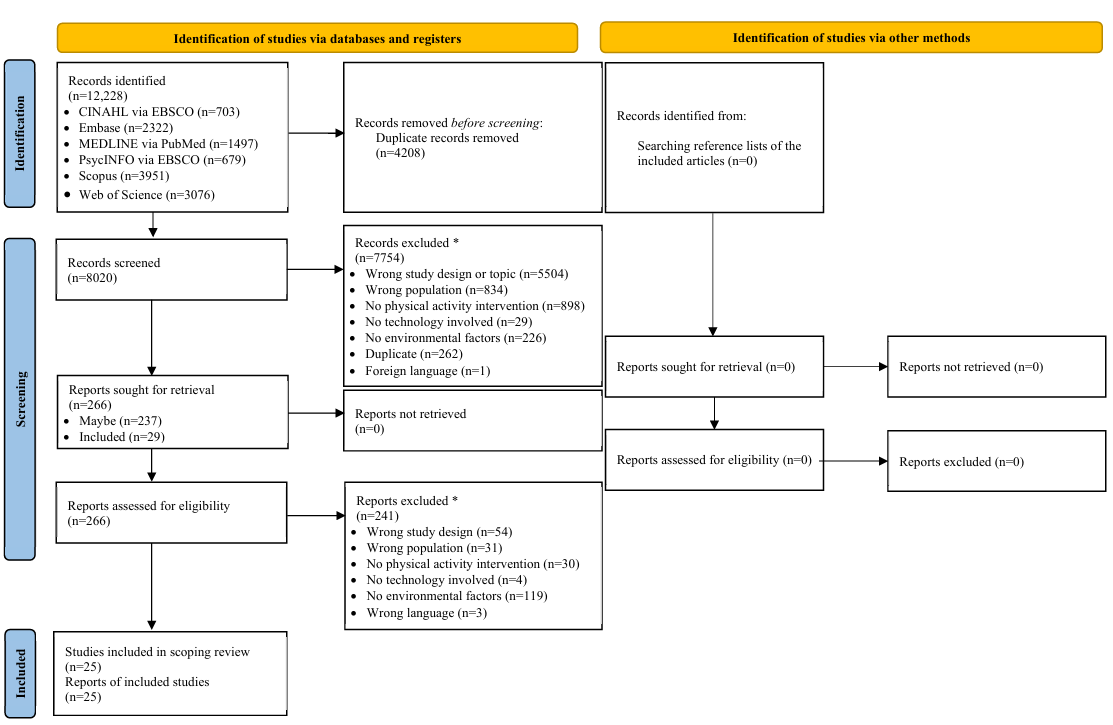
Flow chart of study inclusion. The exclusion of reports was conducted based on the order of the reasons listed in this figure, and only 1 reason for exclusion was recorded.

A total of 12,228 articles were identified from CINAHL (n=703, 5.75%), Embase (n=2322, 18.99%), MEDLINE (n=1497, 12.24%), PsycINFO (n=679, 5.55%), Scopus (n=3951, 32.31%), and Web of Science (n=3076, 25.16%). After removing 34.41% (4208/12,228) of duplicates, 65.59% (8020/12,228) of the articles were included in the title and abstract screening phase. After this phase, of the 8020 included articles, 266 (3.32%) were screened for eligibility in the full-text screening phase. In total, 25 articles met the inclusion criteria and were included in this scoping review (Figure 1) [30-54].

### Study Characteristics

An overview of the characteristics of all 25 included studies is presented in Table 1. The papers were published between 2011 and 2024, and most articles (15/25, 60%) were published between 2019 and 2024. Of the included studies, most (11/25, 44%) were conducted in Europe [33,36,37,39,41,44,45, 47,50-52], followed by North America (5/25, 20%) [32,42,46,49,53], Asia (5/25, 20%) [30,34,35,43,48], and Oceania (4/25, 16%) [31,38,40,54]. The 2 study designs that were most often used across the studies were pretest-posttest (12/25, 48%) [30,32,33,36,41,43,44,46,48,50,52,54] and randomized (controlled) trials (8/25, 32%) [31,34,36-39,41,46]. The sample sizes in the included studies ranged from 1 to 409 (mean 85.7, SD 98.9 participants), and the percentage of women in the study samples ranged from 0% to 100%. Across all the included studies, the lowest and highest reported age of an individual was 50 and 99 years, respectively. The PA components that were most often considered in the technology-assisted PA interventions were (treadmill) walking (8/25, 32%) [30,36,40,42,45,49,53,54] and balance and coordination exercises (8/25, 32%) [34,35,38,44,48,51-53], followed by stretching and flexibility (7/25, 28%) [31,33,34,40,43,50,51] and functional training (5/25, 20%) [33,34,36,51,54]. Of the 25 included studies, 18 (72%) reported supervised PA interventions [34-45,47-49,51,53,54], 4 (16%) included a nonsupervised PA intervention [31,48,52,54], and 3 (12%) did not provide information regarding the supervision of the technology-assisted PA intervention [30,32,33]. The duration of these PA interventions, the frequency and duration of individual intervention sessions, and the technology device used varied across the studies (Table 1).

**Table 1:** Characteristics of the study design, the study sample, and the technology-assisted physical activity interventions in the included studies (N=25).

Study, year, and country	Main study objective	Study design	Sample size, n (% women)	Age (y)	Physical activity component	Technology device	Supervised intervention and duration of the intervention	Session details
Abe et al [30], 2023, Japan	To examine the effects of the Kikoeru app on social connectedness, subjective health, loneliness, and setting a target number of steps	Pretest-posttest without a control group	7 (0)	Mean 74 (SD 4; range 65-78)	Walking	Smartphone	NR^a^ 60 d	Daily use of the app
Alley et al [31], 2024, Australia	To examine the moderating effect of social support on the effectiveness of a web-based, computer-tailored physical activity intervention for older adults	RCT^b^	243 (78.6)	Mean 68.84 (SD 3.85; range 65-98)	Physical activity advice, stretching and flexibility, and strength exercises	Computer and Fitbit device	No 12 wk	Average time on the website during the whole intervention period ranged from 126.89 to 140.24 minutes between groups
Anderson-Hanley et al [32], 2011, United States	To examine the effect of virtual social facilitation (avatars) and competitiveness on exercise effort in exergaming older adults	Pretest-posttest without a control group	14 (92.9)	Total sample: NR (range 60-99); low-competitiveness group: mean 80.7 (SD 12.3; range NR); high-competitiveness group: mean 75.6 (SD 13.5; range NR)	Cycling	Computer	NR 1 mo	2 to 3 rides per week
Boekhout et al [33], 2019, the Netherlands	To examine (1) which individual characteristics predict differences in preference between printed and web-based delivery and (2) which user characteristics and delivery aspects predicted attrition	Pretest-posttest without a control group	409 (64.5)	Total sample: NR; printed delivery group: mean 79.2 (SD 7.6); web-based delivery group: mean 73.3 (SD 6.6)	Functional training and stretching and flexibility	Computer	NR 3 mo	NR
Chen et al [34], 2019, China	To examine the effects of a home-based exercise intervention to reduce knee osteoarthritis symptoms and improve physical functioning in older adults	Quasi-experimental study with a control group	171 (84.4)	Mean 68.9 (SD 7.4)	Functional training, stretching and flexibility, and balance and coordination exercises	Phone	Yes 12 wk	4 sessions of 2 hours; at least 3 sessions of 30-40 minutes per week
Choi and Lee [35], 2019, South Korea	To examine the effects of virtual kayak paddling exercises using real-world video recordings on postural control, muscle performance, and cognitive function in older adults with mild cognitive impairment	RCT	60 (85)	Total sample: NR (range 69-85); intervention group: mean 77.3 (SD 4.4; rage NR); control group: mean 75.4 (SD 4.0; range NR)	Interval training and balance and coordination exercises	Video projected on a screen	Yes 6 wk	2 sessions of 60 minutes per week
Domingos et al [36], 2022, Portugal	To examine the acceptability and safety of delivering dual-task programs in an online group format with people with Parkinson disease in early to late stages of the disease	Pretest-posttest with a control group	15 (60)	Mean 69.4 (SD 9.3; range NR)	Walking and functional training	NR	Yes 16 wk	2 sessions of 60 minutes
Dommes and Cavallo [37], 2012, France	To examine the effects of a training method combined with behavioral and educational interventions on street-crossing decisions by providing practice on a simulator	RCT	40 (57)	Mean 72.2 (SD 5.3; range NR)	Overall physical activity	Simulation laboratory	Yes 1 wk	2 sessions
Duque et al [38], 2013, Australia	To examine the effect of a virtual reality system to assess balance and provide a training system for balance in a population of community-dwelling older participants with a known history of falls	RCT	60 (total sample: NR; intervention group: 63; control group: 61)	Total sample: NR; intervention group: mean 79 (SD 10; range NR); control group: mean 75 (SD 8; range NR)	Balance and coordination exercises	Virtual reality system combining input from a force platform and virtual reality glasses containing a head tracker	Yes 6 wk	2 sessions of 30 minutes per week
Haeger et al [39], 2021, Germany	To examine the effects of a multicomponent approach on walking parameters and assess transfer effects on aspects of cognition, motivation, and control beliefs	RCT	34 (44.1)	Mean 75.0 (SD 3.7; range NR)	Various smartphone-based activities	Smartphone	Yes NR	2 sessions per week
Jansons et al [40], 2017, Australia	To compare the effects on outcome measures of gym-based exercise versus home-based exercise with telephone follow-up among adults with chronic conditions who had completed a short-term exercise program supervised by a health professional	RCT	105 (64)	Total sample: NR; group at home: mean 66 (SD 13; range NR); group at the gym: mean 68 (SD 11; range NR)	Walking, running, weight training, stretching and flexibility, and cycling	Phone	Yes 12 mo	3 sessions of 60 minutes per week
Jurkeviciute et al [41], 2020, Italy and Sweden	To identify contextual factors that determine similarities and differences in the value of an eHealth intervention between 2 contexts	Pretest-posttest without a control group	Italy: 53 (51); Sweden: 54 (56)	Italy: mean 77.6 (SD 5.3); Sweden: mean 74.8 (SD 5.9)	The Otago fall prevention program	Tablet	Yes 6 mo	NR
King et al [42], 2020, United States	To examine whether counseling by a computer-based virtual advisor was not worse than counseling by trained human advisors for increasing 12-month walking levels among inactive older adults	Single-blind, cluster-randomized noninferiority parallel trial	245 (78.8)	Mean 62.3 (SD 8.4; range 50-87)	Walking	Computer	Yes 12 mo	Weekly sessions in the first 2 months, and twice-per-month sessions for the remaining 10 months
Masumoto et al [43], 2017, Japan	To quantitatively measure and visualize face-to-face interactions among older adults in an exercise program and examine relationships among interactional variables; personality; and interest in community involvement, including interactions with the local community	Pretest-posttest without a control group	27 (63)	Mean 73.5 (SD NR; range NR)	Stretching and flexibility and yoga	Karaoke-on-demand system with images projected on a screen	Yes 2 mo	4 sessions of 90 minutes
Nikitina et al [44], 2018, Russia	To examine the feasibility of home-based online group training under different group cohesion settings and its effects on adherence and well-being among older adults; in addition, to assess the effects of a technology-supported intervention on subjective well-being and loneliness	Pretest-posttest without a control group	60 (total sample: NR; pilot study 1: 95; pilot study 2: 100)	Total sample: NR (range 59-83); pilot study 1, individual group: mean 65 (SD 6; range NR); pilot study 1, interaction group: mean 68 (SD 8; range NR); pilot study 2, individual group: mean 69 (SD 7; NR); pilot study 2, interaction group: mean 68 (SD 6; NR)	Weight training and balance and coordination exercises	Tablet and activity monitor	Yes 8 wk	3 sessions of 20-40 minutes per week
O’Brien et al [45], 2021, Ireland	To investigate the experiences and attitudes of older adults following a community-led walking program using activity trackers	Qualitative study	11 (100)	NR (range 60-80)	Walking	Wearable device	Yes 6 wk	Biweekly sessions
Pauly et al [46], 2019, Canada	To examine associations between portable ICT^c^ use and changes in physical activity, loneliness, and executive functioning in older adults	Pretest-posttest without a control group	92 (64)	Mean 67.7 (SD 8.7; range 51-85)	Reporting daily physical activity	Tablet	No 5 wk	NR
Pischke et al [47], 2022, Germany	To compare the acceptance and effectiveness of 2 interventions for physical activity promotion among initially inactive community-dwelling older adults	Cross-over randomized trial	242 (66.2)	Mean 68.7 (SD 5.4; range 60-82)	Physical activity exercises, recommendations, and brochures	Smartphone	Yes 10 wk	1 session of 90 minutes per week
Qiu et al [48], 2023, China	To examine the effects of the Social Balance Ball exergame on intergenerational interactions and assess which factors affect intergenerational interactions in social balance training games	Pretest-posttest	36 (18 older adults; 72.2)	Older adults: mean 64.9 (SD 3.5; range NR)	Balance exercises	Balance board, Social Balance ball, television, and computer	Yes NR	NR
Richards et al [49], 2018, Canada	To show that virtual reality technology can be coupled with a self-paced treadmill to further improve walking competency in individuals after stroke	Single-case study	1 (0)	62	Treadmill walking	Virtual reality–coupled treadmill system	Yes 3 wk	9 sessions in 3 weeks
Scase et al [50], 2017, United Kingdom	To design a gamified environment through which applications could be delivered to promote cognition, exercise, social interaction, and healthy eating and examine adherence to this technology solution through an intervention in which older people were asked to play serious games	Pretest-posttest without a control group	18 (78.6)	Total sample: NR; focus group 1: mean 77.0 (SD 7.5); focus group 2: mean 74.6 (SD 5.5); focus group 3: mean 78.5 (SD 1.9)	Stretching and flexibility	Tablet	No 7 wk	5 sessions per week; the average duration of the sessions was 28 minutes
Thiel et al [51], 2022, Germany	To examine the feasibility and effects of an intervention on combining smartphone-assisted group activities in the neighborhood with training in physical and cognitive skills on the social participation and connectedness of older adults	Noncontrolled proof-of-concept study	39 (85)	Total sample: mean 73.1 (SD 6.8; range NR); cycle group 1: mean 71.9 (SD 7.1; range NR); cycle group 2: mean 73.6 (SD 6.3; range NR)	Aerobic training, functional training, balance and coordination exercises, and stretching and flexibility	Smartphone	Yes 6 mo	1 mandatory session of 90 minutes per week plus additional nonmandatory activities
van Het Reve et al [52], 2014, Switzerland	To compare 3 different home-based training programs and their effects on measures of gait quality while considering adherence to the training program	Pretest-posttest preclinical exploratory trial	44 (NR)	Mean 75 (SD 6; NR)	Strength exercises and balance and coordination exercises	Tablet	No NR	2 sessions of resistance training and 5 sessions of 3 balance exercises
VanRavenstein and Davis [53], 2018, United States	To increase older adults’ daily physical activity with the aim of decreasing chronic disease morbidity, disability, falls, and social isolation	Qualitative study	21 (90)	Total sample: NR (range 57-85); Garden Vistas group: mean 72.8 (SD 9.7; range 58-83); Garden North group: mean 72.3 (SD 7.9; range 57-85)	Walking, step climbing, and balance and coordination exercises	Wearable activity monitor	Yes 12 wk	2 sessions per week
Wilczynska et al [54], 2021, Australia	To conduct a pilot evaluation of the Ecofit intervention using a scalable implementation model among inactive older adults residing in an Australian rural community; to examine the preliminary effectiveness and feasibility of the Ecofit intervention in a “real-world” setting	Pretest-posttest	59 (95)	Mean 62.3 (SD 11.6; range 50-82)	Walking, jogging, outdoor exercises, functional training, and aerobic training	Smartphone	Yes 20 wk	Sessions of 90 minutes

^a^NR not reported.

^b^RCT: randomized controlled trial.

^c^ICT: information and communications technology.

### Environmental Factors and Their Role in Technology-Assisted PA Interventions

An overview of the environmental factors and their role in technology-assisted PA interventions for older adults is presented in Table 2. Of the 25 included studies, 3 (12%) included aspects of multiple environmental domains [41,47,51]. The environmental domain that was most often considered in the included studies was the social environment (19/25, 76%) [30-34,36,40-48,50-53], followed by the physical environment (8/25, 32%) [35,37-39,47,49,51,54], the socioeconomic environment (1/25, 4%) [41], and the systemic environment (1/25, 4%) [41].

Several specific social environmental factors were considered in the various technology-assisted PA interventions across the studies, including social connectedness [30,48,51] (including loneliness [30,33,46]), social interaction [34,43,50,52,53], social support [31,33,36,44,45,47], and delivery aspects (eg, home environment vs gym environment or the involvement of virtual advisors vs human advisors) [32,40,42]. Furthermore, a variety of specific physical environmental factors were also considered, including aspects of the street-crossing environment (eg, traffic speed) [37,49] and the neighborhood built environment (eg, walking paths; presence of benches; and hot spots, ie, highly frequented and meaningful nearby places, such as weekly markets and parks) [38,39,47,49,51,54]. A range of social (eg, household composition), socioeconomic (eg, costs of treatments), and systemic (eg, local preferences on the quality of patient care) environmental factors were considered in one study [41]. In total, 16% (4/25) of the studies were conducted in a simulated or virtual reality environment and included aspects of the physical environment [35,37,38,49]. The tools used to assess the environmental factors differed across the studies (Table 2).

The specific environmental factors fulfilled various roles in the technology-assisted PA interventions in the included studies. A total of 32% (8/25) of the studies included an environmental factor as an outcome. Of these 8 studies, 7 (88%) focused on a social environmental factor [30,34,36,43,45,46,48] and 1 (12%) focused on a physical environmental factor [39]. In 28% (7/25) of the studies, social [40,42,48,51] and physical [35,37,38,47] environmental factors were used as a comparator of the intervention; that is, different environmental backgrounds were compared, such as a virtual program and a face-to-face program, without direct measurement of environmental factors. In another 32% (8/25) of the studies, environmental factors were used as study results or factors influencing the intervention [31,42,44,46,50,51,53,54]. In 12% (3/25) of the studies, social [32,33] and physical [49] environmental factors were used as components of the intervention.

**Table 2:** An overview of technology-assisted physical activity interventions, the role of environmental factors, and outcomes in the included studies (N=25).

Study	Physical activity component and specific technology components	Environmental factor category, role of environmental factor, and specific environmental factor	Assessment tool of environmental factor	Outcomes	Outcome measurements	Main study results	Findings on environmental factors
Abe et al [30], 2023	Walking Kikoeru app on smartphone	Social Outcome Social connectedness and loneliness	Qualitative interviews on social connectedness through app use	Social connectedness related to interactions through the app, subjective health, loneliness, and setting a target number of steps	Qualitative interviews on social connectedness and step goals; subjective health was measured using a VAS^a^, and loneliness was measured using the Ando-Osada-Kodama Loneliness Scale	Social connectedness benefits were reported, loneliness decreased for 4/7 of the participants and remained stable for 3/7, target number of steps increased in 3/7 of the participants and remained stable in 4/7, and the intervention improved subjective health.	Participants experienced social connectedness and reduced loneliness after the intervention.
Alley et al [31], 2024	Physical activity advice, stretching and flexibility, and strength exercises Web-based program with 6 modules of computer-tailored physical activity advice and a Fitbit device	Social Moderator Social support	Abbreviated Duke Social Support Index	Moderate to vigorous physical activity, engagement, and acceptability	ActiGraph GT9X wrist-worn accelerometer, website data and Google Analytics, and 9-item questionnaire	In participants with lower social support, both tailoring-only and Fitbit+tailoring participants increased their moderate to vigorous physical activity from baseline to the postintervention time point, whereas the control group decreased their physical activity. In comparison, all participants with higher social support regardless of group decreased their moderate to vigorous physical activity per day from baseline to the postintervention time point.	Among participants with lower social support, the Fitbit+tailoring participants but not the tailoring-only participants increased their moderate to vigorous physical activity more than the controls. Among participants with higher social support, no differences in moderate to vigorous physical activity changes were observed between groups. No significant (interaction) effects of social support and group were found on engagement and acceptability.
Anderson-Hanley et al [32], 2011	Cycling Cybercycle and computer	Social Social aspect was introduced as an intervention component Virtual social facilitation	NR^b^	Pedaling effort	Cycling exercise effort (watts) captured in 10-second intervals by cybercycle sensors	Significant group (high vs low competitiveness) × time (before to after the introduction of the virtual avatar competitors) interaction; the virtual avatar increased exercise effort among high-competitiveness exercisers	Virtual social facilitation through introduction of avatar competitors increased exercise effort among more competitive exercisers.
Boekhout et al [33], 2019	Functional training and stretching and flexibility Active Plus65 intervention—web based (website) and print based (delivered by mail)	Social Social aspect is part of the intervention components Social support for physical activity and loneliness	Social support for physical activity—self-report (2 questions); loneliness—6-item De Jong Gierveld Loneliness Scale	Delivery mode preference and attrition	Delivery mode and attrition	Attrition differed significantly between the delivery modes—50% in the printed delivery mode and 71% in the web-based delivery mode.	Age and degree of loneliness were significant predictors of delivery mode preference. When adjusting for psychosocial variables, loneliness became nonsignificant, and social support for physical activity then emerged as a significant predictor with participants in the web-based delivery group who had higher levels of social support than those in the printed delivery group.
Chen et al [34], 2019	Functional training, stretching and flexibility, and balance and coordination exercises Telephone support	Social Secondary outcome Social interaction	AIMS2-SF^c^ society dimension	Primary: pain intensity and joint stiffness related to knee osteoarthritis; secondary: muscle strength of the lower limbs, balance, walking ability, and quality of life	Western Ontario and McMaster Universities Osteoarthritis Index	Pain and stiffness had a significantly stronger decrease in the intervention group than in the control group.	Quality of life and the society domain improved in the intervention group but not in the control group.
Choi and Lee [35], 2019	Interval training and balance and coordination exercises Video projected on a screen	Physical Virtual environment was compared to normal home exercise Virtual kayak paddling and home environment	NR	Primary: static and dynamic postural balance; secondary: arm curl test, handgrip strength, and cognitive function	Static balance: 1-leg stance test and the Good Balance System; dynamic balance: Timed Up and Go Test, functional reach test, Berg Balance Scale, and Four Square Step Test	Significant improvement in balance components, motor capacity and function, and cognitive function in the virtual kayak paddling exercise group compared to the control group	Virtual kayak paddling was beneficial for balance, cognition, and muscle performance.
Domingos et al [36], 2022	Walking and functional training Online program (PD3 Move)	Social Outcome Support from family members or caregivers during the sessions	Structured phone interview	Primary: attendance rate and satisfaction with the program; secondary: willingness to attend future online classes, perceived benefits of the program, feedback on format and delivery, and perceived difficulties and facilitators	Exit questionnaire sent via email to participants	Attendance rate and satisfaction were high.	Receiving support from family members or caregivers during the sessions was identified as a facilitator by participants.
Dommes and Cavallo [37], 2012	Overall physical activity Simulation tool in simulation laboratory adapted to street-crossing situation	Physical Setting of the experiment Street-crossing environment	NR	Street-crossing behavior	8 measures describing street-crossing behavior (eg, median accepted time gap between vehicles and collision)	Intervention group showed improvement in street-crossing decisions in (1-week) posttest assessment compared to controls. Differences disappeared 6 months after training.	On both postintervention tests (ie, 1 week and 6 months), the intervention and control groups still made more unsafe decisions when the car was approaching at a high speed and missed more crossing opportunities when a car was approaching at a low speed.
Duque et al [38], 2013	Balance and coordination exercises Balance training protocol with virtual reality system combining input from a force platform and virtual reality glasses containing a head tracker	Physical Setting of the experiment Virtual training versus usual care (including optional Otago participation)	NR	Postural control, falls, fear of falling, gait, serum measurement, depression, and nutrition status	Posturography, retrospective questionnaire on falls, Survey of Activities and Fear of Falling in the Elderly, GAITRite assessment, venous blood, Geriatric Depression Scale, and Mini-Nutritional Assessment	Balance parameters were significantly improved in the BRU^d^ training group. This effect was also associated with a significant reduction in falls and lower levels of fear of falling.	Significantly higher reduction in falls and fear of falling, improvement in balance parameters, and higher adherence to virtual training in the virtual training group compared to the control group.
Haeger et al [39], 2021	Various smartphone-based activities App on smartphone	Physical Outcome Walking path and maximum distance from home	uFall smartphone app	Functional mobility, cognition outcomes, motivation, activity-related outcomes, and personality outcomes	6-Minute Walk Test, Instrumented Timed Up and Go Test, self-concordance and personality, System Usability Scale, Stroop test, and task-switching paradigm	No significant effects on any of the outcomes	No significant effect on Global Position System–based measures
Jansons et al [40], 2017	Walking, running, weight training, stretching and flexibility, and cycling Telephone support	Social Comparison of different environments in which the intervention was carried out Home environment and gym environment	NR	Primary: quality of life; secondary: productivity, social activity, depression and anxiety, motor capacity, physical activity, and attendance to community-based fitness center	Primary: EQ-5D; secondary: Health and Labour Questionnaire, Friendship Scale, Hospital Anxiety and Depression Scale, Phone-FITT^e^, 6-Minute Walk Test, BMI, and 15-second sit-to-stand test	There were no significant differences between study groups in quality of life across the 12-month intervention period. The gym group showed slightly fewer symptoms of depression over the 12-month period than the home group.	No change was found in social isolation.
Jurkeviciute et al [41], 2020	The Otago fall prevention program Web-based portal on a tablet	Social, socioeconomic, and systemic Contextual factors moderating the intervention outcomes Lifestyle habits of the population (eg, if they were living alone or with family), hourly rates of staff for delivering the intervention, organizational setup of the intervention, and local preferences on the quality of patient care	Semistructured interviews (patients and health care professionals) and monetary data from health care and technology providers	Cognitive performance, anxiety, perceived health care satisfaction, and monetary and nonmonetary benefits and sacrifices	Mini-Mental State Examination and the clock-drawing test, EQ-5D-5L, and VAS; other data from semistructured interviews and monetary data from health care and technology providers	In Sweden, patients improved cognitive performance, experienced a reduction in anxiety, and perceived their health as better, and both patients and health care professionals were satisfied with care. There were increased costs and higher workload for health care professionals. The intervention was not cost-efficient. In Italy, patients were satisfied with care, and the health care professionals felt empowered and had an acceptable workload. The intervention was cost-effective. There were no improvements in clinical efficacy and quality of life.	In total, 6 factors that influence eHealth interventions were identified: process of delivery, organizational structure and professionals involved, cost of different treatments, hourly rates of staff for delivering the intervention, lifestyle habits of the population, and local preferences on the quality of patient care.
King et al [42], 2020	Walking Program on computer	Social Comparison of different modes of delivery Virtual advisor and human advisor	NR	Primary: total walking time; secondary: moderate to vigorous physical activity, sedentary behavior, BMI, resting blood pressure and heart rate, and well-being	CHAMPS^f^ questionnaire and Vitality Plus Scale	The 12-month change in walking was more pronounced in the virtual advisor cohort compared to the human advisor cohort. There were improvements in both arms regarding clinical risk factors, sedentary behavior, and well-being.	The virtual advisor produced significant 12-month walking increases for older, low-income Latino adults that were no worse than the significant improvements achieved with human advisors.
Masumoto et al [43], 2017	Stretching and flexibility and yoga The DK Elder System, with a karaoke-on-demand system with images projected on a screen	Social Primary and secondary outcomes Communication networks of participants in the exercise program, time of interactions, number of persons interacted with, interaction among inhabitants, and community involvement	Business Microscope for the primary outcome and environmental factors survey for the secondary outcome	Primary: communication networks of participants in the exercise program, time of interactions, and number of persons interacted with; secondary: interaction among inhabitants and community involvement	Business Microscope (ie, name tag–type wearable sensor node with a built-in infrared signal transmitter and receiver to collect data on the face-to-face interactions of participants)	Network density in the initial session was low but increased as the number of sessions increased. Density in the third session was greater than in the ﬁnal session (ie, increasing the number of sessions does not necessarily lead to promotion of more face-to-face interactions).	Significant enhancement of interest in interacting with local community residents. Marginally significant enhancement of interest in community involvement, communication time, and number of communication partners.
Nikitina et al [44], 2018	Weight training and balance and coordination exercises App; Gymcentral app program; Otago exercise program on a tablet and activity monitor	Social Moderator Social support	Medical Outcomes Study Social Support Survey	Primary: usability and acceptance of the program; secondary: adherence to the program	Usability: System Usability Scale; acceptance: questionnaire	Online group exercising was proven feasible among healthy, independently living older adults in Russia.	Physical training performed in a virtual environment positively affected life satisfaction but not loneliness. High-cohesion groups were preferable for group exercise, and social support was a good predictor for adherence in the low-cohesion condition.
O’Brien et al [45], 2021	Walking Program and wearable device	Social Outcome Views on the social dimension of the program	Interview	Program as a source of motivation, user experiences with the technology, and views on the social dimension of the program	Qualitative interview	The program and activity trackers were useful in maintaining motivation to stay active. Social support was considered a useful component.	Social support was considered a useful component.
Pauly et al [46], 2019	Reporting daily physical activity App (iDialogPad) on tablet	Social Social loneliness as an outcome and social function as a moderator Social loneliness and social function	Social loneliness: revised UCLA^g^ Loneliness Scale; social function: list of ICT^h^ functions including social function	Changes in physical activity, loneliness, and executive functioning	International Physical Activity Questionnaire, revised UCLA Loneliness Scale, and Trail Making Test—part B	No change in physical activity over ≥6 months; time spent sitting decreased. More frequent exercise was associated with more moderate physical activity intensity and less sitting. More frequent use of the social component was associated with more social loneliness.	More frequent use of the social component was associated with more social loneliness of the participants.
Pischke et al [47], 2022	Physical activity exercises, recommendations, and brochures App on smartphone and Fitbit device	Physical and social Background variable Physical activity neighborhood environment, neighborhood environment, walking environment, and social support for engaging in physical activity	Physical activity neighborhood environment scale, neighborhood scales, walking environment, activity-related support from family and friends (modified), and activity-related social support	Primary: moderate to vigorous physical activity and sedentary behavior; secondary: subjective health and technology commitment, use, and experience	Triaxial accelerometers (ActiGraph GT3X+), SF-12^i^ (1 item), and self-generated items	Moderate to vigorous physical activity increased between baseline and T1 (if unadjusted) and decreased between baseline and T2 regardless of the intervention group. A total of 18.6% of the participants met physical activity recommendations at baseline, 16.4% met physical activity recommendations at T1, and 20.3% met physical activity recommendations at T2. For sedentary behavior, there were no significant differences or effects at T1 or T2. Intervention acceptance was high.	NR
Qiu et al [48], 2023	Balance exercises Exergame, balance board, Social Balance Ball, television, and computer	Social Outcome Social networks—social presence and connectedness	Networked Minds Social Presence Inventory, Inclusion of Other in the Self Scale, and Lubben Social Network Scale	Mode of exergames (social presence), role of participants (older or younger), and gender	Mode of exergames and demographic information	Higher levels of social interaction and positive feelings in player modes with human interaction compared to the virtual player mode without human interaction	Social interaction within player modes was associated with more positive experiences as opposed to modes in which no interaction was possible.
Richards et al [49], 2018	Treadmill walking Virtual reality–coupled treadmill system	Physical Part of the intervention Street crossing, corridor walking, park stroll, terrain changes, and moving obstacles	Pictures, virtual reality training settings, and Assessment of Life Habits scale mobility score	Walking competence	5-Meter Walk Test, 6-Minute Walk Test, Berg Balance Scale, Activities-Speciﬁc Balance Conﬁdence Scale, Assessment of Life Habits scale, and personal appraisal	Control protocol training and virtual reality training resulted in a similar progression through the training sessions of total time walked on the treadmill. Virtual reality training led to additional increase in gait speed and 6-Minute Walk Test distance as well as improved balance self-efﬁcacy and anticipatory locomotor adjustments.	Virtual reality training was superior to the control protocol training for improving motor capacity, balance self-efﬁcacy, and anticipatory locomotor adjustments.
Scase et al [50], 2017	Stretching and flexibility App (DOREMI) on tablet	Social The social interaction element enhanced well-being Social interaction	Thematic analysis for social aspects	Adherence to the intervention	Number of sessions and mean session length	Significant group differences in engagement in game sessions related to different social arrangements. Gamified environment can help engage with computer-based applications. Social community factors influenced long-term adherence.	Social community factors influenced adherence to the intervention. Bonding and sense of community between participants supported engagement.
Thiel et al [51], 2022	Aerobic training, functional training, balance and coordination exercises, and stretching and flexibility App (designed for the Quartier Agil program) on smartphone	Physical and social Facilitator Hot spots (highly frequented and meaningful sites) in the neighborhood environment	Hot spots were identified in group discussions; social participation facet of the World Health Organization Quality of Life Instrument–Older Adults	Physical function, balance, leg strength, and physical activity level	6-Minute Walk Test, Berg Balance Scale, isometric leg strength, and sensor-based moderate to vigorous physical activity	Combined physical and cognitive training supported by technical devices (smartphones) appears feasible.	The hot spots can be considered a facilitator of the intervention.
van Het Reve et al [52], 2014	Strength exercises and balance and coordination exercises Active Lifestyle app on tablet	Social Social aspect in one group compared to other intervention modalities Social interaction	Number of dispatched messages to a bulletin board (within the social group only)	Gait analysis, physical performance, fear of falling, and adherence	Walking analysis (GAITRite), Short Physical Performance Battery, Falls Efficacy Scale International, and compliance recordings	Tablet groups showed significant improvements in gait parameters and adherence compared to the brochure group but not in physical performance.	Program adherence was highest in the group with a social aspect compared to other intervention modalities.
VanRavenstein and Davis [53], 2018	Walking, step climbing, and balance and coordination exercises Telehealth, exercise program (Otago), and Fitbit device	Social Moderator of intervention effect Social isolation and depression	Qualitative analysis to form categories about socialization	Mobility	Self-Efficacy for Exercise Scale, 30-second sit-to-stand test, Mini-Balance Evaluation Systems test, Berg Balance Scale, and 2-Minute Walk Test	Successful implementation of telehealth physical therapy–led intervention to increase physical activity. Social isolation and depression need to be addressed to encourage successful aging in place.	Physical activity and socialization are critical to older adults who are aging in place. Mental health needs to be considered when attempting to engage older adults in group activities.
Wilczynska et al [54], 2021	Walking, jogging, outdoor exercises, functional training, and aerobic training Ecofit app on smartphone	Physical Facilitator of the intervention Outdoor built environmental characteristics (ie, railings, stairs, benches, and parks)	No assessment; built environment was used in the intervention.	Aerobic fitness, functional mobility, and upper- and lower-body muscular fitness	6-Minute Walk Test, Timed Up and Go Test, arm curl test, and chair stand test	Significant improvements in aerobic fitness, functional mobility, and upper- and lower-body muscular fitness at 6 and 20 weeks.	The Ecofit program makes use of simple infrastructure (ie, railings, stairs, and benches) and can be adapted to outdoor locations.

^a^VAS: visual analog scale.

^b^NR: not reported.

^c^AIMS2-SF: Arthritis Impact Measurement Scales 2–Short Form.

^d^BRU: balance rehabilitation unit.

^e^Phone-FITT: brief physical activity interview for older adults.

^f^CHAMPS: Community Health Activities Model Program for Seniors.

^g^UCLA: University of California, Los Angeles.

^h^ICT: information and communications technology.

^i^SF-12: 12-item Short Form Health Survey.

### Intervention Outcomes by Environmental Factor

As described in Table 2, the technology-assisted PA interventions in most studies (19/25, 76%) were evaluated positively [30,32-38,41-43,45,48-54]. The included environmental factors played a supportive role in achieving this effect in several of these studies in which a between-group comparison was made [33-38,42]. In some studies, no effects (3/25, 12%) [31,39,40], mixed effects (2/25, 8%) [44,47], or adverse effects (1/25, 4%) of the interventions or environmental factors (eg, moderators) were reported [46].

The studies that considered social environmental factors in the technology-assisted PA interventions (12/25, 48%) focused on a variety of outcomes, including PA and exercise [30-32,46]; mental health outcomes [30,32,46]; quality of life [34,40]; physical performance [52,53]; physical health outcomes [42]; social interaction network [43]; and aspects such as delivery, acceptability, usability, adherence, attrition, satisfaction, experiences, and motivation [31,33,36,44,45,48,50,52]. The studies that considered physical environmental factors (7/25, 28%) also focused on various outcomes, including cognition [35,39], physical performance [35,38,39,49,51,54], adherence [38], and street-crossing decision-making [37]. The 12% (3/25) of the studies that considered aspects of multiple environmental domains also focused on various outcomes, including PA [47,51]; mental health [41,47]; cognition [41]; physical performance [51]; costs [41]; and aspects such as satisfaction, commitment, experience, and acceptability [47].

In several studies (5/25, 20%), the technology-assisted PA interventions improved social environmental factors, such as social connectedness [30], social interaction networks [43], and other societal factors [34]. In one study, the technology-assisted PA intervention did not change social isolation in older adults [40]. In the study by Pauly et al [46], more frequent use of the social component of an app was associated with more loneliness in older adults. In various studies (7/25, 28%), social interaction and support were found to increase the exercise effort in more competitive but not in less competitive older adults [32], as well as adherence [36,50,52,53] and the positive experiences [48] of older adults in technology-assisted PA interventions. In contrast, in the study by Alley et al [31], older adults with lower baseline levels of social support increased their moderate to vigorous PA more than those with higher levels of social support.

In various studies (5/25, 20%), physical environmental factors were considered in a virtual or simulated environment [35,37,38,49]. Compared to usual home exercises, virtual kayak paddling was beneficial for balance, muscle performance, and cognition [35]. In a simulated street-crossing situation, the intervention group improved street-crossing decisions compared to controls, but these differences disappeared 6 months after training [37]. On postintervention tests, the intervention group and the control group both made more unsafe decisions when a car approached at a high speed and missed more crossing opportunities when a car was approaching at a low speed compared to the preintervention test [37]. In the study by Duque et al [38], there were significantly fewer falls and lower levels of fear of falling in older adults who received balance training in a virtual environment compared to a control group that received usual care. The study by Richards et al [49] showed that virtual reality training was superior to the control protocol training for improving motor capacity, balance self-efficacy, and anticipatory locomotor adjustments.

One study showed that a technology-assisted PA intervention that made use of features of the outdoor built environment (community hot spots such as parks and markets) improved physical function in older adults [51]. Through the definition of hot spots in the neighborhood, older adults were encouraged to carry out certain activities with their peers when meeting there. However, other technology-assisted PA interventions that considered such physical environmental factors did not significantly affect functional mobility [39] or only showed limited effectiveness on PA outcomes [47]. The study by Jurkeviciute et al [41] indicated that several social, socioeconomic, and systemic environmental factors influenced the effects of an eHealth intervention focusing on cognitive performance, mental health outcomes, satisfaction, and costs.

## Discussion

### Principal Findings

#### Overview

With this work, we provide an overview of the role of environmental factors in technology-assisted PA interventions for the target group of older adults. It has been suggested previously that environmental aspects play a crucial role in modulating human behavior, especially regarding PA [55]. Emerging evidence has shown that PA should not be seen as an isolated entity but—apart from being an intrinsically motivated behavior—also as an output and reaction to environmental circumstances and surroundings [56]. These surroundings can have many shapes as they can be differentiated in many ways. In this work, we chose to partition the potentially numerous environmental influences into physical, social, socioeconomic, and systemic environmental factors. This provided the opportunity for a comprehensive description of a variety of aspects that may (or may not) interact with technology-assisted interventions to increase or optimize PA in older adults. The fact that 25 studies from almost all continents were identified highlights that researchers worldwide are aware of the relevance of environmental factors in such settings. However, this number of studies is not high, which indicates that this topic is still emerging. As expected, these studies were highly variable in terms of design, sample size, and intervention characteristics.

#### Social Environment

The social environment was the most frequently addressed environmental domain as it was considered in 76% (19/25) of the studies [30-34,36,40-48,50-53]. A total of 37% (7/19) of these studies used aspects of the social environment as an outcome [30,34,36,43,45,46,48]. The main reason for this probably lies in the (partially empirically substantiated) expectation that PA is related to increased social participation [20] and reduced loneliness [57]. However, whether this is the case would need further empirical substantiation. Previous research has shown that PA interventions per se do not necessarily come with the advantage of additional social benefits [58], but technological solutions might add benefits related to (online) connectedness and communication options. Mixed results on social environmental factors were found, showing benefits for social connectedness [30], social interaction networks [43], and other societal factors [44] but not for social isolation [40,46]. An important social aspect seems to be social comparison or role models, which may have been the reason for increased exercise effort [32] and adherence [36,50,52]. However, it has to be acknowledged that social comparison was not assessed as such in these studies. Nonetheless, it may be that, through seeing peers performing well or better than oneself, people tend to put more effort into their exercise and PA behavior [59]. Furthermore, social support has been shown to be important for adherence and maintaining motivation [44,45] and to be a significant predictor of delivery preference (ie, people who receive higher social support for PA were more likely to prefer a web-based delivery than a printed delivery) [33]. However, there were also results showing that lower social support at baseline may come with more room for benefits in moderate to vigorous PA when applying tailored online exercise advice [31]. The study by King et al [42] suggests that delivery aspects can also affect intervention outcomes. In this study, counseling by virtual advisors significantly increased walking in low-income, Latino older adults, which was comparable to the significant improvements achieved with human advisors [43].

#### Physical Environment

The second most considered environmental domain was the physical environment (8/25, 32% of the studies) [35,37-39,47,49,51,54]. In 50% (4/8) of these studies, physical environmental factors were considered in a virtual or simulated environment [35,37,38,49]. It is an important finding that physical and cognitive capacities such as balance, muscle performance, and cognition can be enhanced when exercising under these virtual or simulated environmental conditions [35,37,38,49]. These effects even translated to a reduction in the number of falls and fear of falling in one study compared to controls who received usual care [38]. There seems to be a huge potential and growing empirical foundation for using augmented and virtual reality–based exercise applications [60]. The constant development and sophistication of these technologies in terms of physical experience and aspects of (online) communication highlight the manifold potential for future application in research and beyond. However, there are several barriers to the use of such technologies when targeting older adults that may hamper their success [61]. Beyond virtual environments, physical environmental factors from the “real world” were also considered, mainly concerning the neighborhood built environment (eg, walking paths, hot spots, and benches) [38,39,47,49,51,54].

#### Socioeconomic and Systemic Environment

An investigation of socioeconomic and systemic environmental factors was carried out in the study by Jurkeviciute et al [41], where both aspects were considered as moderators of the intervention effect. These authors found several factors that influence eHealth intervention effects, including the process of delivery, organizational structure and professionals involved, treatment costs including hourly rates of staff for delivering the intervention, lifestyle habits of the population, and local preferences regarding quality of patient care [41]. While these findings are from one study only and have to be interpreted with caution, they do underline that intervention effects often are a product of their setting and the professionals (eg, trainers and therapists) and participants involved. More research is needed on the role of socioeconomic and systemic environmental factors in technology-assisted PA interventions to draw more concrete conclusions on this issue.

### Implications for Future Research, Practice, and Policy

On the basis of our findings, it becomes apparent that there is a significant potential for a better understanding of intervention results and better tailoring of intervention programs when including environmental aspects in research endeavors. Environmental factors demonstrated multifaceted effects on intervention outcomes, albeit sometimes contradictory. A thorough understanding of the underlying mechanisms is still lacking as the use and applications of these environmental factors remain scarce. Future research should elucidate the causal pathways through which environmental factors exert their effects, considering potential mediators and moderators that may influence intervention outcomes. In particular, social aspects related to intervention delivery and group dynamics seem to have the potential to reveal important mechanisms that could positively enhance technology-assisted PA interventions for older adults. The same applies to the physical environment. We found that physical and even cognitive capacity may benefit largely from exercise in virtual or simulated environments, showing the large potential for augmented and virtual reality exercises. As this is a field growing at high speed, further work should be carried out to define more specific methodologies and target outcomes especially for the population of older adults. Considering that young and middle-aged adults are using these kinds of technologies more and more, the potential for interventions targeted at PA will be enormous. As such technologies become increasingly accessible and user-friendly, their integration into PA interventions holds promise for enhancing engagement and adherence and obtaining positive outcomes among older adults. As older adults are not left out of these developments and show more acceptance of such technologies [62], intervention development can be expected to be more inclusive of virtual environments in the future.

### Strengths and Limitations

We followed a well-defined methodology for this scoping review. Although we conducted a detailed search in various established databases, we might have missed potentially relevant papers (eg, from gray literature or conference proceedings not yet published as full papers). It also has to be acknowledged that there is no clear framework for categorizing environmental factors into specific domains (ie, social, physical, socioeconomic, and systemic). The framework we decided to use was based on thorough discussions within the group of researchers involved. We chose not to evaluate the quality of the studies included in this scoping review as the objective of this work was to explore the role of environmental factors in technology-assisted PA interventions among older adults and not to assess the quality of the studies. As a result, the study findings need to be interpreted with caution, especially those of studies without randomization procedures or controls. The comprehensive summary of methods in Table 1 provides all the information in this regard. As technology-assisted PA interventions can be expected to grow in number and application in the following years, evaluating the quality of the evidence is undoubtedly warranted at some point in the future.

### Conclusions

In conclusion, the results of this scoping review addressing the fast-growing domain of technology-assisted PA interventions for older adults show that the role of environmental factors is still emerging in this field. The studies predominantly focused on social environmental factors, followed by physical environmental factors. Studies that integrate socioeconomic and systemic environmental factors in technology-assisted PA interventions were scarce. Important findings were that the included environmental factors played a supportive role in achieving beneficial effects of technology-assisted PA interventions. The studies reviewed exhibited heterogeneity in how environmental factors were incorporated—some studies incorporated them as integral components of the experimental design, whereas in other studies, they served as effect modifiers or outcomes. Drawing from the results, there is a significant potential for a better understanding of intervention outcomes and better tailoring of intervention programs when systematically including environmental aspects in technology-assisted PA interventions for older adults.

## Appendix

Multimedia Appendix 1 PRISMA-ScR (Preferred Reporting Items for Systematic Reviews and Meta-Analyses extension for Scoping Reviews) checklist.

Multimedia Appendix 2 Detailed search strategy for each bibliographic database.

## Abbreviations

PA: physical activity
PRISMA-ScR: Preferred Reporting Items for Systematic Reviews and Meta-Analyses Extension for Scoping Reviews
