# Correction: The Role of Environmental Factors in Technology-Assisted Physical Activity Intervention Studies Among Older Adults: Scoping Review

**DOI:** 10.2196/83822

**Published:** 2025-09-18

**Authors:** Carl-Philipp Jansen, Désirée Nijland, Jean-Michel Oppert, Veysel Alcan, Kirsi E Keskinen, Emmi Matikainen-Tervola, Zada Pajalic, Merja Rantakokko, Signe Tomsone, Essi-Mari Tuomola, Erja Portegijs, Erik J Timmermans

**Affiliations:** 1 Geriatric Center Medical Faculty Heidelberg Heidelberg University Heidelberg Germany; 2 Clinic for Geriatric Rehabilitation Robert Bosch Hospital Stuttgart Germany; 3 Department of Human Movement Sciences University Medical Center Groningen University of Groningen Groningen The Netherlands; 4 Department of Nutrition Pitié-Salpêtrière Hospital (AP-HP) Sorbonne University Paris France; 5 Department of Electrical and Electronics Engineering Engineering Faculty Tarsus University Tarsus Turkey; 6 Gerontology Research Center and Faculty of Sport and Health Sciences University of Jyväskylä Jyväskylä Finland; 7 Institute of Rehabilitation JAMK University of Applied Sciences Jyväskylä Finland; 8 Campus Drammen, Faculty of Health and Social Sciences University of South-Eastern Norway Oslo Norway; 9 The Wellbeing Services County of Central Finland Jyväskylä Finland; 10 Department of Rehabilitation Faculty of Health and Sport Sciences Rīga Stradiņš University Riga Latvia; 11 Julius Center for Health Sciences and Primary Care University Medical Center Utrecht Utrecht University Utrecht The Netherlands

In “The Role of Environmental Factors in Technology-Assisted Physical Activity Intervention Studies Among Older Adults: Scoping Review” (JMIR Mhealth Uhealth 2025;13(1):e59570) the authors noted one error.

The first sentence of the acknowledgement was an error, stating:

“This scoping review is being undertaken as part of the European Cooperation in Science and Technology Action “PhysAgeNet” (CA20104).”

This sentence has been corrected to:

“This publication is based upon work from COST Action CA20104 – Network on evidence-based physical activity in old age (PhsAgeNet), supported by COST (European Cooperation in Science and Technology).”

The correction will appear in the online version of the paper on the JMIR Publications website, together with the publication of this correction notice. Because this was made after submission to PubMed, PubMed Central, and other full-text repositories, the corrected article will also be resubmitted to those repositories.

