# Supplementary material for: The Role of Environmental Factors in Technology-Assisted Physical Activity Intervention Studies Among Older Adults: Scoping Review
Source: JMIR Mhealth Uhealth. 2025 Mar 13;13:e59570. doi: 10.2196/59570 (PMC11950703; doi:10.2196/59570)
Supplement: Multimedia Appendix 2 [file mhealth_v13i1e59570_app2.docx]

**Multimedia Appendix 2: Detailed search strategy for each bibliographic database**

**Search strategy for CINAHL (via EBSCO)**

| **Topic blocks** | **Terms** | **#** |
| --- | --- | --- |
| 1. Intervention type | AB (exercis* OR recreation OR physical activit* OR sport* OR “physical training” OR “strength training” OR “resistance training” OR “balance training” OR “aerobic training” OR “anaerobic training" OR “endurance training” OR “muscle training” OR “functional training” OR “multi-component training” OR “circuit training” OR “serious gam*” OR exergam* OR “wii training” OR “kinect training” OR intervention) OR TI (exercis* OR recreation OR physical activit* OR sport* OR “physical training” OR “strength training” OR “resistance training” OR “balance training” OR “aerobic training” OR “anaerobic training" OR “endurance training” OR “muscle training” OR “functional training” OR “multi-component training” OR “circuit training” OR “serious gam*” OR exergam* OR “wii training” OR “kinect training” OR intervention) OR (MH “Exercise”) OR (MH “Exergaming”) | #1 |
| 2. Technology | AB (ehealth OR mhealth OR e-health OR m-health OR technology-assisted OR apps OR “mobile app*” OR ICT OR “web-based” OR “online intervention” OR “online training” OR “online program*” OR remote OR smartphone OR “smart phone” OR tracker* OR sensor OR “mobile applications” OR “virtual reality” OR “internet-based intervention”) OR TI (ehealth OR mhealth OR e-health OR m-health OR technology-assisted OR apps OR “mobile app*” OR ICT OR “web-based” OR “online intervention” OR “online training” OR “online program*” OR remote OR smartphone OR “smart phone” OR tracker* OR sensor OR “mobile applications” OR “virtual reality” OR “internet-based intervention”) OR (MH “Telemedicine” OR (MH “Digital Technology”) | #2 |
| 3. Target group | AB (“Older adult” OR “older adults” OR seniors OR elder* OR “older men” OR “older women” OR “older people” OR “older individuals” OR old-age OR “older age” OR “older person” OR seniors) OR TI (“Older adult” OR “older adults” OR seniors OR elder* OR “older men” OR “older women” OR “older people” OR “older individuals” OR old-age OR “older age” OR “older person” OR seniors) OR (MH “Geriatrics”) | #3 |
| 4. Environment | AB (Country OR culture OR “political climate” OR “health policy” OR climate OR demography OR ethnicity OR race OR racial OR governance OR macroeconomic policies OR social policies OR public policies OR cultural values OR societal values  OR “Social Participation” OR “Social Isolation” OR “social support” OR “social network” OR “social contact” OR “social interaction” OR social disadvantage OR social class OR social cohesion OR social capital OR social relationships OR safety OR social norms OR social values OR buddy OR friend OR family OR “crime rate” OR “poverty rate” OR “socioeconomic status” OR “socio-economic status” OR education OR “educational level” OR income OR “area deprivation” OR occupation OR “built environment” OR “physical environment” OR “objective environment” OR environment OR “home environment” OR GPS OR life-space OR “life space” OR “activity space” OR activity-space OR “urban form” OR “urban design” OR neighborhood OR neighbourhood OR GIS OR “geographic information system” OR “open space” OR “greenspace” OR “green space” OR “blue space” OR pedestrian-friendly OR walkability OR walkab* OR bikeab* OR cyclab* OR park OR parks OR “living environment” OR “land use” OR “land cover” OR “land use mix” OR “environmental indicators” OR “street connectivity” OR greenness OR “traffic density” OR “retail density” OR “service density“ OR “recreational spaces” OR “population density” OR “urbanization grade” OR “sidewalk density” OR “public transport” OR “sports facilities”) OR TI (Country OR culture OR “political climate” OR “health policy” OR climate OR demography OR ethnicity OR race OR racial OR governance OR macroeconomic policies OR social policies OR public policies OR cultural values OR societal values OR “Social Participation” OR “Social Isolation” OR “social support” OR “social network” OR “social contact” OR “social interaction” OR social disadvantage OR social class OR social cohesion OR social capital OR social relationships OR safety OR social norms OR social values OR buddy OR friend OR family OR “crime rate” OR “poverty rate” OR “socioeconomic status” OR “socio-economic status” OR education OR “educational level” OR income OR “area deprivation” OR occupation OR “built environment” OR “physical environment” OR “objective environment” OR environment OR “home environment” OR GPS OR life-space OR “life space” OR “activity space” OR activity-space OR “urban form” OR “urban design” OR neighborhood OR neighbourhood OR GIS OR “geographic information system” OR “open space” OR “greenspace” OR “green space” OR “blue space” OR pedestrian-friendly OR walkability OR walkab* OR bikeab* OR cyclab* OR park OR parks OR “living environment” OR “land use” OR “land cover” OR “land use mix” OR “environmental indicators” OR “street connectivity” OR greenness OR “traffic density” OR “retail density” OR “service density“ OR “recreational spaces” OR “population density” OR “urbanization grade” OR “sidewalk density” OR “public transport” OR “sports facilities”) OR (MH “Social Inclusion”) OR (MH “Social Isolation”) OR (MH "Social Environment") OR (MH “Socioeconomic Factors”) OR MH ("Environment") OR (MH "Health Facility Environment") OR (MH "Environment Design") OR (MH "Home Environment") OR (MH "Built Environment") OR (MH “Residence Characteristics”) OR (MH "Sports and Recreational Facilities") | #4 |
| Final search string | #1 AND #2 AND #3 AND #4 | #5 |

**Search strategy for EMBASE**

| **Topic blocks** | **Terms** | **#** |
| --- | --- | --- |
| 1. Intervention type | exercis*:ab,ti OR recreation:ab,ti OR 'physical activit*':ab,ti OR sport*:ab,ti OR 'physical training':ab,ti OR 'strength training':ab,ti OR ‘resistance training’:ab,ti OR ‘balance training’:ab,ti OR ‘aerobic training’:ab,ti OR ‘anaerobic training’:ab,ti OR ‘endurance training’:ab,ti OR ‘muscle training’:ab,ti OR ‘functional training’:ab,ti OR ‘multi-component training’:ab,ti OR ‘circuit training’:ab,ti OR ‘serious gam*’:ab,ti OR exergam*:ab,ti OR ‘wii training’:ab,ti OR ‘kinect training’:ab,ti OR intervention:ab,ti OR ‘Exercise’/exp OR ‘Exergaming’/exp | #1 |
| 2. Technology | ehealth:ab,ti OR mhealth:ab,ti OR e-health:ab,ti OR m-health:ab,ti OR technology-assisted:ab,ti OR apps:ab,ti OR ‘mobile app*’:ab,ti OR ICT:ab,ti OR ‘web-based’:ab,ti OR ‘online intervention’:ab,ti OR ‘online training’:ab,ti OR ‘online program*’:ab,ti OR remote:ab,ti OR smartphone:ab,ti OR ‘smart phone’:ab,ti OR tracker*:ab,ti OR sensor:ab,ti OR ‘mobile applications’:ab,ti OR ‘virtual reality’:ab,ti OR ‘internet-based intervention’:ab,ti OR ‘Telemedicine’/exp OR ‘Digital Technology’/exp | #2 |
| 3. Target group | ‘Older adult’:ab,ti OR ‘older adults’:ab,ti OR seniors:ab,ti OR elder*:ab,ti OR ‘older men’:ab,ti OR ‘older women’:ab,ti OR ‘older people’:ab,ti OR ‘older individuals’:ab,ti OR old-age:ab,ti OR ‘older age’:ab,ti OR ‘older person’:ab,ti OR seniors:ab,ti OR ‘Geriatrics’/exp | #3 |
| 4. Environment | Country:ab,ti OR culture:ab,ti OR ‘political climate’:ab,ti OR ‘health policy’:ab,ti OR climate:ab,ti OR demography:ab,ti OR ethnicity:ab,ti OR race:ab,ti OR racial:ab,ti OR governance:ab,ti OR ‘macroeconomic policies’:ab,ti OR ‘social policies’:ab,ti OR ‘public policies’:ab,ti OR ‘cultural values’:ab,ti OR ‘societal values’:ab,ti OR ‘Social Participation’:ab,ti OR ‘Social Isolation’:ab,ti OR ‘social support:ab,ti’ OR ‘social network’:ab,ti OR ‘social contact’:ab,ti OR ‘social interaction’:ab,ti OR ‘social disadvantage’:ab,ti OR ‘social class’:ab,ti OR ‘social cohesion’:ab,ti OR ‘social capital’:ab,ti OR ‘social relationships’:ab,ti OR safety:ab,ti OR ‘social norms’:ab,ti OR ‘social values’:ab,ti OR buddy:ab,ti OR friend:ab,ti OR family:ab,ti OR ‘crime rate’:ab,ti OR ‘poverty rate’:ab,ti OR ‘socioeconomic status’:ab,ti OR ‘socio-economic status’:ab,ti OR education:ab,ti OR ‘educational level’:ab,ti OR income:ab,ti OR ‘area deprivation’:ab,ti OR occupation:ab,ti OR ‘built environment’:ab,ti OR ‘physical environment’:ab,ti OR ‘objective environment’:ab,ti OR environment:ab,ti OR ‘home environment’:ab,ti OR GPS:ab,ti OR life-space:ab,ti OR ‘life space’:ab,ti OR ‘activity space’:ab,ti OR activity-space:ab,ti OR ‘urban form’:ab,ti OR ‘urban design’:ab,ti OR neighborhood:ab,ti OR neighbourhood:ab,ti OR GIS:ab,ti OR ‘geographic information system’:ab,ti OR ‘open space’:ab,ti OR ‘greenspace’:ab,ti OR ‘green space’:ab,ti OR ‘blue space’:ab,ti OR pedestrian-friendly:ab,ti OR walkability:ab,ti OR walkab*:ab,ti OR bikeab*:ab,ti OR cyclab*:ab,ti OR park:ab,ti OR parks:ab,ti OR ‘living environment’:ab,ti OR ‘land use’:ab,ti OR ‘land cover’:ab,ti OR ‘land use mix’:ab,ti OR ‘environmental indicators’:ab,ti OR ‘street connectivity’:ab,ti OR greenness:ab,ti OR ‘traffic density’:ab,ti OR ‘retail density’:ab,ti OR ‘service density’:ab,ti OR ‘recreational spaces’:ab,ti OR ‘population density’:ab,ti OR ‘urbanization grade’:ab,ti OR ‘sidewalk density’:ab,ti OR ‘public transport’:ab,ti OR ‘sports facilities’:ab,ti OR ‘Social Inclusion’/exp OR ‘Social Isolation’/exp OR ‘Social Environment’/exp OR ‘Socioeconomic Factors’/exp OR ‘Environment’/exp OR ‘Health Facility Environment’/exp OR ‘Environment Design’/exp OR ‘Home Environment’/exp OR ‘Built Environment’/exp OR ‘Residence Characteristics’/exp OR ‘Sports and Recreational Facilities’/exp | #4 |
| Final search string | #1 AND #2 AND #3 AND #4 | #5 |

**Search strategy for MEDLINE (via Pubmed):**

| **Topic blocks** | **Terms** | **#** |
| --- | --- | --- |
| 1. Intervention type | exercis*[tiab] OR recreation[tiab] OR physical activit*[tiab] OR sport*[tiab] OR “physical training”[tiab] OR “strength training”[tiab] OR “resistance training”[tiab] OR “balance training”[tiab] OR “aerobic training”[tiab] OR “anaerobic training"[tiab] OR “endurance training”[tiab] OR “muscle training”[tiab] OR “functional training”[tiab] OR “multi-component training”[tiab] OR “circuit training”[tiab] OR “serious gam*”[tiab] OR exergam*[tiab] OR “wii training”[tiab] OR “kinect training”[tiab] OR intervention[tiab] OR exercise[mesh] OR exergaming[mesh:noexp] | #1 |
| 2. Technology | ehealth[tiab] OR mhealth[tiab] OR e-health OR m-health OR technology-assisted[tiab] OR apps[tiab] OR “mobile app*”[tiab] OR ICT[tiab] OR “web-based”[tiab] OR “online intervention”[tiab] OR “online training”[tiab] OR “online program*”[tiab] OR remote[tiab] OR smartphone[tiab] OR “smart phone”[tiab] OR tracker*[tiab] OR sensor[tiab] OR “mobile applications”[tiab] OR “virtual reality”[tiab] OR “internet-based intervention”[mesh] OR telemedicine[mesh] OR “digital technology”[mesh] | #2 |
| 3. Target group | “Older adult”[tiab] OR “older adults”[tiab] OR seniors[tiab] OR elder*[tiab] OR “older men”[tiab] OR “older women”[tiab] OR “older people”[tiab] OR “older individuals”[tiab] OR old-age[tiab] OR “older age”[tiab] OR “older person”[tiab] OR seniors[tiab] OR geriatrics[mesh] | #3 |
| 4a. Systemic environment | Country[tiab] OR culture[tiab] OR “political climate”[tiab] OR “health policy” OR climate[tiab] OR demography[tiab] OR ethnicity[tiab] OR race[tiab] OR racial[tiab] OR governance[tiab] OR macroeconomic policies[tiab] OR social policies[tiab] OR public policies[tiab] OR cultural values[tiab] OR societal values[tiab] | #4 |
| 4b. Social environment | “Social Participation”[tiab] OR “Social Isolation”[tiab] OR “social support”[tiab] OR “social network”[tiab] OR “social contact”[tiab] OR “social interaction”[tiab] OR social disadvantage[tiab] OR social class[tiab] OR social cohesion[tiab] OR social capital[tiab] OR social relationships[tiab] OR safety[tiab] OR social norms[tiab] OR social values[tiab] OR buddy[tiab] OR friend[tiab] OR family[tiab] OR “Social Inclusion”[mesh] OR “Social Isolation”[mesh] OR "Social Environment"[mesh] | #5 |
| 4c. Socioeconomic environment | “crime rate”[tiab] OR “poverty rate”[tiab] OR “socioeconomic status”[tiab] OR “socio-economic status”[tiab] OR education[tiab] OR “educational level”[tiab] OR income[tiab] OR “area deprivation”[tiab] OR occupation OR “socioeconomic factors”[mesh] | #6 |
| 4d. Physical environment | “built environment”[tiab] OR “physical environment”[tiab] OR “objective environment”[tiab] OR environment[tiab] OR “home environment”[tiab] OR GPS[tiab] OR life-space[tiab] OR “life space”[tiab] OR “activity space”[tiab] OR activity-space[tiab] OR “urban form”[tiab] OR “urban design”[tiab] OR neighborhood[tiab] OR neighbourhood[tiab] OR GIS[tiab] OR “geographic information system”[tiab] OR “open space”[tiab] OR “greenspace”[tiab] OR “green space”[tiab] OR “blue space”[tiab] OR pedestrian-friendly[tiab] OR walkability[tiab] OR walkab*[tiab] OR bikeab*[tiab] OR cyclab*[tiab] OR park[tiab] OR parks[tiab] OR “living environment”[tiab] OR “land use”[tiab] OR “land cover”[tiab] OR “land use mix”[tiab] OR “environmental indicators”[tiab] OR “street connectivity”[tiab] OR greenness[tiab] OR “traffic density”[tiab] OR “retail density”[tiab] OR “service density“[tiab] OR “recreational spaces”[tiab] OR “population density”[tiab] OR “urbanization grade”[tiab] OR “sidewalk density”[tiab] OR “public transport”[tiab] OR “sports facilities”[tiab] OR "Environment"[Mesh] OR "Health Facility Environment"[Mesh] OR "Environment Design"[Mesh] OR "Home Environment"[Mesh] OR "Built Environment"[Mesh] OR “residence characteristics”[Mesh] OR "Sports and Recreational Facilities"[Mesh] | #7 |
| Final search string | #1 AND #2 AND #3 AND (#4 OR #5 OR #6 OR #7) | #8 |

**Search strategy for PsycInfo (via EBSCO)**

| **Topic blocks** | **Terms** | **#** |
| --- | --- | --- |
| 1. Intervention type | AB (exercis* OR recreation OR physical activit* OR sport* OR ‘physical training’ OR ‘strength training’ OR ‘resistance training’ OR ‘balance training’ OR ‘aerobic training’ OR ‘anaerobic training’ OR ‘endurance training’ OR ‘muscle training’ OR ‘functional training’ OR ‘multi-component training’ OR ‘circuit training’ OR ‘serious gam*’ OR exergam* OR ‘wii training’ OR ‘kinect training’ OR intervention) OR TI (exercis* OR recreation OR physical activit* OR sport* OR ‘physical training’ OR ‘strength training’ OR ‘resistance training’ OR ‘balance training’ OR ‘aerobic training’ OR ‘anaerobic training’ OR ‘endurance training’ OR ‘muscle training’ OR ‘functional training’ OR ‘multi-component training’ OR ‘circuit training’ OR ‘serious gam*’ OR exergam* OR ‘wii training’ OR ‘kinect training’ OR intervention) OR (DE ‘Exercise’) OR (DE ‘Exergaming’) | #1 |
| 2. Technology | AB (ehealth OR mhealth OR e-health OR m-health OR technology-assisted OR apps OR ‘mobile app*’ OR ICT OR ‘web-based’ OR ‘online intervention’ OR ‘online training’ OR ‘online program*’ OR remote OR smartphone OR ‘smart phone’ OR tracker* OR sensor OR ‘mobile applications’ OR ‘virtual reality’ OR ‘internet-based intervention’) OR TI (ehealth OR mhealth OR e-health OR m-health OR technology-assisted OR apps OR ‘mobile app*’ OR ICT OR ‘web-based’ OR ‘online intervention’ OR ‘online training’ OR ‘online program*’ OR remote OR smartphone OR ‘smart phone’ OR tracker* OR sensor OR ‘mobile applications’ OR ‘virtual reality’ OR ‘internet-based intervention’) OR (DE ‘Telemedicine’ OR (DE ‘Digital Technology’) | #2 |
| 3. Target group | AB (‘Older adult’ OR ‘older adults’ OR seniors OR elder* OR ‘older men’ OR ‘older women’ OR ‘older people’ OR ‘older individuals’ OR old-age OR ‘older age’ OR ‘older person’ OR seniors) OR TI (‘Older adult’ OR ‘older adults’ OR seniors OR elder* OR ‘older men’ OR ‘older women’ OR ‘older people’ OR ‘older individuals’ OR old-age OR ‘older age’ OR ‘older person’ OR seniors) OR (DE ‘Geriatrics’) | #3 |
| 4 Environment | AB (Country OR culture OR ‘political climate’ OR ‘health policy’ OR climate OR demography OR ethnicity OR race OR racial OR governance OR macroeconomic policies OR social policies OR public policies OR cultural values OR societal values  OR ‘Social Participation’ OR ‘Social Isolation’ OR ‘social support’ OR ‘social network’ OR ‘social contact’ OR ‘social interaction’ OR social disadvantage OR social class OR social cohesion OR social capital OR social relationships OR safety OR social norms OR social values OR buddy OR friend OR family OR ‘crime rate’ OR ‘poverty rate’ OR ‘socioeconomic status’ OR ‘socio-economic status’ OR education OR ‘educational level’ OR income OR ‘area deprivation’ OR occupation OR ‘built environment’ OR ‘physical environment’ OR ‘objective environment’ OR environment OR ‘home environment’ OR GPS OR life-space OR ‘life space’ OR ‘activity space’ OR activity-space OR ‘urban form’ OR ‘urban design’ OR neighborhood OR neighbourhood OR GIS OR ‘geographic information system’ OR ‘open space’ OR ‘greenspace’ OR ‘green space’ OR ‘blue space’ OR pedestrian-friendly OR walkability OR walkab* OR bikeab* OR cyclab* OR park OR parks OR ‘living environment’ OR ‘land use’ OR ‘land cover’ OR ‘land use mix’ OR ‘environmental indicators’ OR ‘street connectivity’ OR greenness OR ‘traffic density’ OR ‘retail density’ OR ‘service density’ OR ‘recreational spaces’ OR ‘population density’ OR ‘urbanization grade’ OR ‘sidewalk density’ OR ‘public transport’ OR ‘sports facilities’) OR TI (Country OR culture OR ‘political climate’ OR ‘health policy’ OR climate OR demography OR ethnicity OR race OR racial OR governance OR macroeconomic policies OR social policies OR public policies OR cultural values OR societal values OR ‘Social Participation’ OR ‘Social Isolation’ OR ‘social support’ OR ‘social network’ OR ‘social contact’ OR ‘social interaction’ OR social disadvantage OR social class OR social cohesion OR social capital OR social relationships OR safety OR social norms OR social values OR buddy OR friend OR family OR ‘crime rate’ OR ‘poverty rate’ OR ‘socioeconomic status’ OR ‘socio-economic status’ OR education OR ‘educational level’ OR income OR ‘area deprivation’ OR occupation OR ‘built environment’ OR ‘physical environment’ OR ‘objective environment’ OR environment OR ‘home environment’ OR GPS OR life-space OR ‘life space’ OR ‘activity space’ OR activity-space OR ‘urban form’ OR ‘urban design’ OR neighborhood OR neighbourhood OR GIS OR ‘geographic information system’ OR ‘open space’ OR ‘greenspace’ OR ‘green space’ OR ‘blue space’ OR pedestrian-friendly OR walkability OR walkab* OR bikeab* OR cyclab* OR park OR parks OR ‘living environment’ OR ‘land use’ OR ‘land cover’ OR ‘land use mix’ OR ‘environmental indicators’ OR ‘street connectivity’ OR greenness OR ‘traffic density’ OR ‘retail density’ OR ‘service density’ OR ‘recreational spaces’ OR ‘population density’ OR ‘urbanization grade’ OR ‘sidewalk density’ OR ‘public transport’ OR ‘sports facilities’) OR (DE ‘Social Inclusion’) OR (MH ‘Social Isolation’) OR (DE ‘Social Environment’) OR (DE ‘Socioeconomic Factors’) OR (DE ‘Environment’) OR (DE ‘Health Facility Environment’) OR (DE ‘Environment Design’) OR (DE ‘Home Environment’) OR (DE ‘Built Environment’) OR (DE ‘Residence Characteristics’) OR (DE ‘Sports and Recreational Facilities’) | #4 |
| Final search string | #1 AND #2 AND #3 AND #4 | #5 |

**Search strategy for Scopus**

| **Topic blocks** | **Terms** | **#** |
| --- | --- | --- |
| 1. Intervention type | TITLE-ABS-KEY(exercis* OR recreation OR ‘physical activit*’ OR sport* OR ‘physical training’ OR ‘strength training’ OR ‘resistance training’ OR ‘balance training’ OR ‘aerobic training’ OR ‘anaerobic training’ OR ‘endurance training’ OR ‘muscle training’ OR ‘functional training’ OR ‘multi-component training’ OR ‘circuit training’ OR ‘serious gam*’ OR exergam* OR ‘wii training’ OR ‘kinect training’ OR intervention OR exercise OR exergaming) | #1 |
| 2. Technology | TITLE-ABS-KEY(ehealth OR mhealth OR e-health OR m-health OR technology-assisted OR apps OR ‘mobile app*’ OR ICT OR web-based OR ‘online intervention’ OR ‘online training’ OR ‘online program’ OR remote OR smartphone OR ‘smart phone’ OR tracker* OR sensor OR ‘mobile applications’ OR ‘virtual reality’ OR ‘internet-based intervention’ OR telemedicine OR ‘digital technology’) | #2 |
| 3. Target group | TITLE-ABS-KEY(‘Older adult’ OR ‘older adults’ OR seniors OR elder* OR ‘older men’ OR ‘older women’ OR ‘older people’ OR ‘older individuals’ OR old-age OR ‘older age’ OR ‘older person’ OR seniors OR geriatrics) | #3 |
| 4a. Systemic environment | TITLE-ABS-KEY(Country OR culture OR ‘political climate’ OR ‘health policy’ OR climate OR demography OR ethnicity OR race OR racial OR governance OR ‘macroeconomic policies’ OR ‘social policies’ OR ‘public policies’ OR ‘cultural values’ OR ‘societal values’) | #4 |
| 4b. Social environment | TITLE-ABS-KEY(‘Social Participation’ OR ‘Social Isolation’ OR ‘social support’ OR ‘social network’ OR ‘social contact’ OR ‘social interaction’ OR ‘social disadvantage’ OR ‘social class’ OR ‘social cohesion’ OR ‘social capital’ OR ‘social relationships’ OR safety OR ‘social norms’ OR ‘social values’ OR buddy OR friend OR family OR ‘Social Inclusion’ OR ‘Social Isolation’ OR ‘Social Environment’) | #5 |
| 4c. Socioeconomic environment | TITLE-ABS-KEY(‘crime rate’ OR ‘poverty rate’ OR ‘socioeconomic status’ OR ‘socio-economic status’ OR education OR ‘educational level’ OR income OR ‘area deprivation’ OR occupation OR ‘socioeconomic factors’) | #6 |
| 4d. Built environment | TITLE-ABS-KEY(‘built environment’ OR ‘physical environment’ OR ‘objective environment’ OR environment OR ‘home environment’ OR GPS OR life-space OR ‘life space’ OR ‘activity space’ OR activity-space OR ‘urban form’ OR ‘urban design’ OR neighborhood OR neighbourhood OR GIS OR ‘geographic information system’ OR ‘open space’ OR greenspace OR ‘green space’ OR ‘blue space’ OR pedestrian-friendly OR walkability OR walkab* OR bikeab* OR cyclab* OR park OR parks OR ‘living environment’ OR ‘land use’ OR ‘land cover’ OR ‘land use mix’ OR ‘environmental indicators’ OR ‘street connectivity’ OR greenness OR ‘traffic density’ OR ‘retail density’ OR ‘service density’ OR ‘recreational spaces’ OR ‘population density’ OR ‘urbanization grade’ OR ‘sidewalk density’ OR ‘public transport’ OR ‘sports facilities’ OR Environment OR ‘Health Facility Environment’ AND ‘Environment Design’ OR ‘Home Environment’ OR ‘Built Environment’ OR ‘residence characteristics’ OR ‘Sports and Recreational Facilities’) | #7 |
| Final search string | #1 AND #2 AND #3 AND (#4 OR #5 OR #6 OR #7) | #8 |

**Search strategy for Web of Science**

| **Topic blocks** | **Terms** | **#** |
| --- | --- | --- |
| 1. Intervention type | TS=(exercis* OR recreation OR ‘physical activit*’ OR sport* OR ‘physical training’ OR ‘strength training’ OR ‘resistance training’ OR ‘balance training’ OR ‘aerobic training’ OR ‘anaerobic training’ OR ‘endurance training’ OR ‘muscle training’ OR ‘functional training’ OR ‘multi-component training’ OR ‘circuit training’ OR ‘serious gam*’ OR exergam* OR ‘wii training’ OR ‘kinect training’ OR intervention OR exercise OR exergaming) | #1 |
| 2. Technology | TS=(ehealth OR mhealth OR e-health OR m-health OR technology-assisted OR apps OR ‘mobile app*’ OR ICT OR web-based OR ‘online intervention’ OR ‘online training’ OR ‘online program’ OR remote OR smartphone OR ‘smart phone’ OR tracker* OR sensor OR ‘mobile applications’ OR ‘virtual reality’ OR ‘internet-based intervention’ OR telemedicine OR ‘digital technology’) | #2 |
| 3. Target group | TS=(‘Older adult’ OR ‘older adults’ OR seniors OR elder* OR ‘older men’ OR ‘older women’ OR ‘older people’ OR ‘older individuals’ OR old-age OR ‘older age’ OR ‘older person’ OR seniors OR geriatrics) | #3 |
| 4a. Systemic environment | TS=(Country OR culture OR ‘political climate’ OR ‘health policy’ OR climate OR demography OR ethnicity OR race OR racial OR governance OR ‘macroeconomic policies’ OR ‘social policies’ OR ‘public policies’ OR ‘cultural values’ OR ‘societal values’) | #4 |
| 4b. Social environment | TS=(‘Social Participation’ OR ‘Social Isolation’ OR ‘social support’ OR ‘social network’ OR ‘social contact’ OR ‘social interaction’ OR ‘social disadvantage’ OR ‘social class’ OR ‘social cohesion’ OR ‘social capital’ OR ‘social relationships’ OR safety OR ‘social norms’ OR ‘social values’ OR buddy OR friend OR family OR ‘Social Inclusion’ OR ‘Social Isolation’ OR ‘Social Environment’) | #5 |
| 4c. Socioeconomic environment | TS=(‘crime rate’ OR ‘poverty rate’ OR ‘socioeconomic status’ OR ‘socio-economic status’ OR education OR ‘educational level’ OR income OR ‘area deprivation’ OR occupation OR ‘socioeconomic factors’) | #6 |
| 4d. Physical environment | TS=(‘built environment’ OR ‘physical environment’ OR ‘objective environment’ OR environment OR ‘home environment’ OR GPS OR life-space OR ‘life space’ OR ‘activity space’ OR activity-space OR ‘urban form’ OR ‘urban design’ OR neighborhood OR neighbourhood OR GIS OR ‘geographic information system’ OR ‘open space’ OR greenspace OR ‘green space’ OR ‘blue space’ OR pedestrian-friendly OR walkability OR walkab* OR bikeab* OR cyclab* OR park OR parks OR ‘living environment’ OR ‘land use’ OR ‘land cover’ OR ‘land use mix’ OR ‘environmental indicators’ OR ‘street connectivity’ OR greenness OR ‘traffic density’ OR ‘retail density’ OR ‘service density’ OR ‘recreational spaces’ OR ‘population density’ OR ‘urbanization grade’ OR ‘sidewalk density’ OR ‘public transport’ OR ‘sports facilities’ OR Environment OR ‘Health Facility Environment’ AND ‘Environment Design’ OR ‘Home Environment’ OR ‘Built Environment’ OR ‘residence characteristics’ OR ‘Sports and Recreational Facilities’) | #7 |
| Final search string | #1 AND #2 AND #3 AND (#4 OR #5 OR #6 OR #7) | #8 |
